# Early-life Risk Factors for Adult Chronic Disease: Follow-up of a Cohort Born During 1964–1978 in an Urban Slum of Lahore, Pakistan

**Published:** 2008-03

**Authors:** Fehmida Jalil, Sophie E. Moore, Nadeem S. Butt, Rifat N. Ashraf, Shakila Zaman, Andrew M. Prentice, Lars Å. Hanson

**Affiliations:** 1 Department of Social and Preventive Paediatrics, King Edward Medical College and Mayo Hospital, Lahore, Pakistan; 2 MRC International Nutrition Group, London School of Hygiene & Tropical Medicine, London, UK; 3 Department of Social and Preventive Paediatrics, Fatima Jinnah Medical College, Lahore, Pakistan; 4 Department of Clinical Immunology, University of Göteborg, Göteborg, Sweden

**Keywords:** Birthweight, Chronic disease, Follow-up studies, Risk factors, Slums, Pakistan

## Abstract

Evidence suggests that risk of chronic diseases may be programmed during the foetal and early life of the infant. With high rates of low birthweight coupled with a rapid nutritional transition, low-income countries are facing an epidemic of chronic diseases. Follow-up of a cohort of adults born during 1964–1978 in an urban slum in Lahore, Pakistan, is presented in this paper. In 695 of these adults (mean age=29.0 years, males=56%), blood pressure, fasting blood glucose, and body mass index (BMI) were measured to assess early-life predictors of risk of chronic diseases. Sixteen percent of the study population was born with a low birthweight (<2,500 g). A significant positive association (p=0.007) was observed between birthweight and BMI; additionally, adjusting for age and gender, the association with BMI was highly significant (p=0.000). Conversely, a significant negative association (p=0.016) was observed between birthweight and adult levels of fasting plasma glucose; after adjustment for age and gender, the association was more significant (p=0.005) No association was observed between birthweight and adult blood pressure. The results suggest that low birthweight may increase later risk of impaired glucose tolerance in urban Pakistani adults. Further research in this area is warranted.

## INTRODUCTION

The effect that the early-life environment has on later health and survival is presently the subject of much scientific interest and debate, with rates of both pre- and postnatal growth linked to later risk of disease (1,2). A considerable number of studies have demonstrated significant associations among birthweight, rates of growth during foetal life, or early infancy, and a range of risk factors for chronic disease in adulthood, including raised blood pressure (3), impaired glucose tolerance (4,5), dyslipidaemia (6), and obesity (7). These findings have led to the formulation of the ‘developmental origins of health and disease’ (DOHaD) hypothesis.

The vast majority of low-birthweight babies are born in developing countries and, with such high rates of foetal growth restriction, one would presume a high later risk of disease. Arguably however, the foetal origins hypothesis might not be of great significance in such countries if the population remains lean, fit, and frugal (8). However, with increasing affluence and urbanization and the associated transition towards a more Western lifestyle, developing countries now face an increasing risk for chronic disease. These countries might, thus, be at risk from this mismatch between early nutritional deprivation and later nutritional affluence (9).

Although the foetal origins hypothesis has been much debated and frequently re-examined in affluent populations, long-term record-keeping has not been a high priority in developing countries, meaning that the opportunities for follow-up are extremely rare in the very populations, in which the hypothesis may have the greatest public-health significance. The increasing prevalence of obesity, dyslipidaemia, and diabetes mellitus in middle age, particularly in females, needs immediate attention in terms of prevention and health education in such economically-deprived populations (10).

According to the National Health Survey, obesity is more common among females than among males in Pakistan for all age-groups and in both urban and rural areas (10). In most age-groups, levels double comparing rural and urban populations. For example, among the age-group of 25–44 years, 9% of rural males were obese compared to 22% of urban males. Fourteen percent of rural females in the same age-group were obese compared to 37% of urban females. As expected, the prevalence of type 2 diabetes increases with age in Pakistan, from 5% for the age-group of 25–44 years to 12% among those aged over 65 years in rural areas. The highest (20%) prevalence was found among urban females. Hypertension is also highly prevalent among obese Pakistanis, with approximately 58% of overweight males aged 45 years and over being hypertensive (10).

While little previous research has been conducted in Pakistan, the incidences of type 2 diabetes and ischemic heart disease are rising rapidly in neighbouring India (11), coinciding with increasing urbanization and obesity. Indian babies are exceptionally small, with a mean birthweight of only 2,700 g, and 30% have a birthweight of 2,650 g or less (12). Their mothers are short and underweight, with a mean body mass index (BMI) of only 18 kg/m^2^. Furthermore, it has been observed that these small Indian babies have a low muscle mass, small viscera, and a relative excess of fat, a body composition particularly likely to lead to insulin resistance (13).

The current study represents a follow-up of health and survival of a longitudinal cohort of subjects born in a poor urban slum in Lahore, Pakistan, during 1964–1978, with the specific aim of investigating the relationship between early life and later risk of adult diseases. This paper describes the original data-collection protocol together with the results of the recent follow-up study of adult health indicators. It also describes the potential value of this cohort of Pakistani adults for future studies in this area of research.

## MATERIALS AND METHODS

### Infant study, 1964–1978

In the early 1960s, an urban settlement in Lahore, Pakistan, was selected for a community-based follow-up study of infant health (14). At the start of the study, a cross-sectional survey was conducted to register families and to record information on fami-ly size, structure, and socioeconomic status. These subjects came from an urban housing colony, representing a slum area of Gowalmandi, Lahore. In this conservative community, a young female doctor knocking at each door was culturally unacceptable, and working in this environment was difficult. Despite the difficulties, this initial survey provided an opportunity to get acquainted with the families, which facilitated future work. The team of a doctor (FJ), a public-health nurse, and a field assistant, gradually gained the confidence of the community, was accepted over the years and was trusted and welcome in later years of the study.

Approximately 1,000 families enrolled into the study during 16 years and 2,468 babies born into these families were registered. At the time of the study, birth registration with local authorities was mandatory for the traditional birth attendants (TBAs). However, at the start of the project, the rate of registration was quite low and, as such, a monetary incentive was given to the TBAs to encourage the prompt registration of all births. The project team then obtained information about new births through daily visits to the office of the local authority located in the study area. Despite this procedure and the incentive offered, many infants were still not registered immediately and, hence, the recruitment of the infants into the study started at any time between birth and two months of age. Furthermore, during interactions with the families, the team learned that, in the Indo-Pakistan subcontinent, deaths during the traditional ‘*Chilla* period’, when mother and baby remain confined, were concealed from relatives and friends because of superstitions that a spell is cast on the visitor, and the babies born to the visitor will die in the early neonatal period. To avoid social isolation, these births, particularly of low-birthweight babies, and deaths were most often not reported even to the local authorities. However, despite these difficulties, the majority of infants were seen shortly after birth, with nearly 41% seen within one week, 50% within 14 days, 65% within 28 days, and nearly 70% within 60 days of birth. At this first visit, the weight, length, and head circumference were measured using standard equipment. Of these, only the newborns weighed within seven days of birth were included for analysis.

Following the initial visit, the infants were seen each week at home for the first 40 days (*Chilla* period); after this, mothers were encouraged to bring the child to the health clinic monthly until six months, quarterly until two years, and then yearly until 12 years of age. The visits were less frequent after 1–2 year(s), as most often the mother was pregnant again. Nearly 55% of the children were examined 3–9 times during the first two years, while 30% were examined 10–39 times. At each visit, the mother was encouraged to have the child weighed using a beam scale and length measured by a locally-manufactured portable length metre. Milestones of development and history of feeding and illnesses for each child were also recorded. The World Health Organization (WHO) standards were taken for the evaluation of growth (15).

The recruitment into the current project was terminated in 1978 and replaced by a longitudinal study in four different socioeconomic groups from Pakistan (16).

### Follow-up study, 2000–2003

**Fig. 1:**
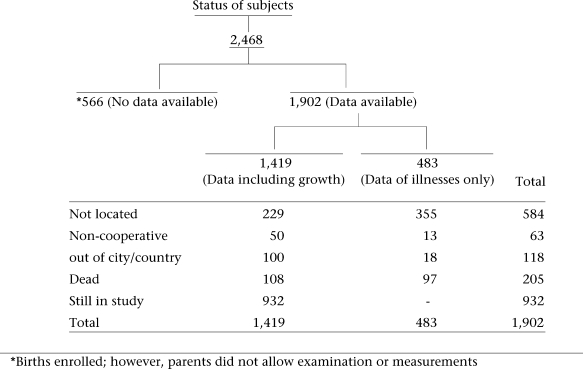
Cohort 1964–1978: status of subjects in the cohort

In view of the increasing interest in the effects that the early-life environment has on later health and survival, a study was designed to retrace this cohort of subjects. During January-June 2000, a team of field staff visited the study families to identify the whereabouts of index subjects. From 2,468 infants registered during the original study, 1,902 (77%) had early-life data available and were considered for follow-up in the present study. Of these, 584 (30.6%) could not be found, 63 (3.3%) refused to participate in the study, and 205 (10.5%) had died since their initial registration, while 118 (6.2%) were either out of the city or out of the country. In total, 932 (49%) adults were successfully recruited into this follow-up study. No attempt was made to trace 566 subjects for whom only socioeconomic data were recorded during infancy; for these subjects, early anthropometric data were not available as the field team was not permitted to measure the newborn. Figure 1 shows the follow-up details for the study cohort.

A new questionnaire was administered to each adult enrolled into the study (n=932) to record the current health status, and a trained doctor conducted a complete physical examination. A Tanita electronic scale (Tanita UK, West Drayton, Middlesex, UK) was used for measuring weight, and height was measured using a portable stadiometre. Blood pressure was measured in triplicate, with subjects seated and at rest, using a sphygmomanometer (Yamasu, wall type, made in Japan) (17). In total, 695 (75%) subjects agreed to give blood samples for evaluation of fasting blood sugar, while the remaining 25% refused. A sample of venous blood was then collected, having ensured that the subject had not had anything to eat or drink during the previous 12–14 hours. The fasting levels of plasma glucose were measured using the enzymatic calorimetric GOD-PAP (Hexokinase) method (18).

The Medical Ethics Committee for Research of King Edward Medical College, Lahore, Pakistan, and the Ethics Committee of University of Gothenburg, Sweden, approved the study.

### Data analysis

Of the 932 subjects traced to follow-up, 695 (74.6%) had fasting blood glucose levels determined. Of these, 583 (83.9%) had a birthweight measured within one week (excluding premature infants and twins) and were, therefore, included in the analysis of the associations between birthweight and adult variables. As mentioned, the infants were measured only when the mothers chose to bring their infants for growth monitoring. Thus, the number of infants measured at each specific age points was limited and varied according to time point.

Simple linear and multiple linear regression models were used for relating the birthweight with adult variables: BMI, blood sugar, and systolic and diastolic blood pressure. Some influential cases were removed from regression analysis based on high leverage values and DFBetas (19,20).

Independent samples *t*-test was used for comparing plasma glucose level in subjects who were short and obese with those who were tall and obese.

Birthweight was used as a continuous variable for exploring associations with the adult variables. Associations of birthweight with the adult variable were analyzed using three Models. Model 1 was without making any adjustments, Model 2 with adjustment with age and gender, and Model 3 with age, gender, and BMI.

Standard deviation (SD) scores were calculated using data from local Pakistani upper-middle class population groups as reference (21). For stunting and wasting, the WHO/United Nations Children's Fund definition of <2 SD score was applied (15).

## RESULTS

### Subject characteristics in infancy

The characteristics of the infants collected during the initial study in 1964–1978 are summarized in the next few paragraphs.

#### Body size at first contact and growth in infancy

Table 1 details anthropometric measures at birth (all infants seen within 7 days of birth), at 8 to 28 days, and at age 12 months and 24 months. The mean birthweight was 3.2 kg for boys and 3 kg for girls, with 16% born <2,500 g and, therefore, classified as low birthweight. At birth, males were heavier and longer than females, and this remained the case up until 12 months of age. There was a decline in nutritional status, with 21% of subjects classified as having moderate to severe wasting and 23% as having moderate to severe stunting at 12 months of age.

**Table 1:** Anthropometric measurements of study population at birth

Body measurements at birth with SDS and SD	Male			Female			Total		
	No.	Mean	SD	No.	Mean	SD	No.	Mean	SD
Birth-7 days									
Weight (kg)	312	3.2	0.6	256	3.0	0.6	568	3.1	0.6
Length (cm)	223	51.3	2.9	225	50.2	2.7	448	50.7	2.9
Head circumference (cm)	209	34.9	1.6	202	34.1	1.5	411	34.5	1.6
SDS weight	312	-0.9	1.1	256	1.0	1.0	568	0.0	1.4
SDS length	223	0.0	1.5	225	−0.3	1.2	448	−0.2	1.4
SDS head circumference	209	−0.3	1.3	202	−0.7	1.2	411	−0.5	1.3
8–28 days									
Weight (kg)	237	3.4	0.6	222	3.3	0.6	459	3.4	0.6
Length (cm)	234	52.7	2.7	219	51.7	2.4	453	52.2	2.6
Head circumference (cm)	231	35.7	1.6	220	35.1	1.6	451	35.4	1.6
SDS weight	237	−0.9	1.0	222	−0.8	1.0	459	−0.9	1.0
SDS length	234	0.1	1.4	219	−0.1	1.1	453	0.0	1.3
SDS head circumference	231	−0.3	1.3	220	−0.4	1.2	451	−0.3	1.3
11–13 months									
Weight (kg)	250	8.6	1.3	216	7.8	1.3	466	8.2	1.4
Length (cm)	227	72.1	3.6	205	70.3	3.8	432	71.2	3.8
Head circumference (cm)	191	45.5	1.9	169	43.9	1.5	360	44.7	1.9
SDS weight	250	−1.0	1.1	216	−1.3	1.3	466	−1.1	1.2
SDS length	227	−1.0	1.3	205	−1.2	1.5	432	−1.1	1.4
SDS head circumference	191	−0.3	1.4	169	−1.7	3.1	360	−1.0	2.5

SD=Standard deviation; SDS=Standard deviation score

#### Infant-feeding practices

Details of infant-feeding practices are shown in Table 2. Rates of breastfeeding were very low, with only 25.9% of the infants being predominantly breastfed by one month of age and with 37% of the subjects receiving no breast milk, being solely bottle-fed from the first week of life.

**Table 2:** Patterns of infant-feeding from first week of life to 12 months

Feeding pattern	1 week	1 month	6 months	9 months	12 months
	(n=325)	(n=436)	(n=695)	(n=574)	(n=590)
Predominantly breastfed	44.6	25.9	6.2	9.9	8.5
Bottle milk	36.9	46.8	26.0	47.0	45.4
Breast milk + bottle milk	18.5	27.3	6.2	8.2	8.6
Semi-solids	0.0	0.0	0.0	13.4	16.3
Bottle milk + semi-solids	0.0	0.0	50.4	14.6	16.1
Breast milk + semi-solids	0.0	0.0	5.7	3.5	2.5
Breast milk + bottle milk + semi-solids	0.0	0.0	5.0	3.5	2.5

Figures shown in percentages

#### Morbidity

**Fig. 2:**
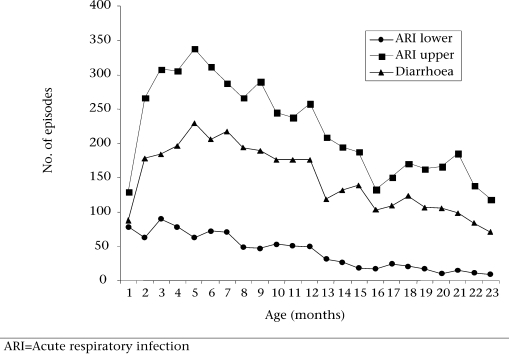
Age-wise distribution of number of episodes of diarrhoea and lower and upper respiratory infections

Figure 2 shows the episodes of lower and upper respiratory infections and diarrhoeal diseases by age. All peaked between two and six months.

#### Mortality

In total, 205 deaths were recorded in the cohort during 1964–2003. Data on mortality were collected using a previously-validated verbal autopsy tool (16). Two doctors used the tool independently, and if the findings agreed, the report was accepted. In the case of disagreement, a third senior doctor conducted the verbal autopsy; the findings were again matched to see if these agreed with any of the previous two and then accepted. Owing to the problems of reaching all births, rates of neonatal mortality were difficult to obtain. As a consequence, these deaths could not be accurately calculated, and this shortcoming makes it difficult to calculate rates of neonatal, infant, and under-five mortality. However, the maximum number of deaths was reported during the first year of life. In this cohort, more male than female deaths were observed in all age-groups, except those who were aged 6–15 years. In the age-group of >15 years, the number of male deaths was more than twice the number of female deaths. This was due to an excess of accident-related deaths in males. There were six deaths due to sudden cardiac failure and another six deaths due to renal failure; four of the deaths due to renal failure had a history of dialysis, one had congenital renal anomaly, and the sixth remained unconfirmed.

### Subject characteristics at follow-up

Characteristics of the subjects by gender, at follow-up are detailed in Table 3. The adult males were significantly heavier and taller than the females, but had very similar mean BMI scores. Using the proposed BMI classification for Pakistanis, 10% were undernourished (below 18.5 kg/m^2^), 25% had average nutritional status (BMI 18.5–22.9 kg/m^2^), 25.2% were overweight and at risk (BMI 23–24.9 kg/m^2^), 29% were obese stage 1 (BMI 25–29.8 kg/m^2^), and 10.8% very obese stage II (BMI >30 kg/m^2^), i.e. nearly 40% were obese.

**Table 3:** Comparison of adult characteristics by gender

Variable	Males (n=392)		Females (n=303)	
	Mean	SD	Mean	SD
Age (years)	29.0	5.5	29.0	5.0
Weight (kg)	70.5	15.5	60.8	22.5
Height (cm)	170.2	7.1	154.9	6.0
BMI (kg/m^2^)	24.2	4.7	25.2	8.8
Systolic BP (mm Hg)	116.5	14.3	110.7	13.8
Diastolic BP (mm Hg)	79.3	9.7	75.1	11.1
Blood glucose (mg/dL)	85.7	19.5	87.4	25.8

BMI=Body mass index; BP=Blood pressure; SD=Standard deviation

The mean systolic and diastolic blood pressure was 116.5 mm Hg and 79.3 mm Hg in males and 110.7 mm Hg and 75.1 mm Hg for females respectively. Both systolic and diastolic blood pressure levels were significantly higher in males than in females. No trend with age was observed in the blood pressure values. Of the 695 adult subjects analyzed, 6.4% had raised systolic blood pressure (≥140 mm Hg), and 16.6 % had raised diastolic blood pressure (≥90 mm Hg).

The mean fasting levels of blood glucose were 85.7 mg/dL for males and 87.4 mg/dL for females. Fasting blood glucose levels were impaired in 43 (6.2%) of the adult subjects (≥110 mg/dL) (4,5), and 22 subjects (3.2%) had a fasting blood glucose level of ≥140 mg/dL and can, therefore, be classified as diabetic (4,5). No trend with age was observed in the blood sugar values.

### Associations between birthweight and adulthood characteristics

Table 4 details the comparison between normal and low birthweight and adult variables.

**Table 4:** Association of adult variables and birthweight

Variable	Coeff.	SE	*t*	p>|*t*|	95% CI	Model significance
Model 1: Dependent variable						
Body mass index						
Constant	21.3	1.2	17.5	0.000	18.92, 23.69	0.0071
Birthweight	1.1	0.4	2.7	0.007	0.29, 1.81	
Model 2: Dependent variable						
Body mass index						
Constant	11.5	2.3	4.9	0.000	6.94, 16.12	0.0000
Birthweight	1.6	0.4	3.6	0.000	0.72, 2.47	
Age	0.2	0.0	4.3	0.000	0.11, 0.31	
Gender	1.4	0.5	2.7	0.008	0.36, 2.40	
Model 1: Dependent variable						
Fasting blood sugar						
Constant	97.4	4.8	20.3	0.000	88.02, 106.88	0.0156
Birthweight	−3.7	1.5	−2.4	0.016	−6.75, −0.71	
Model 2: Dependent variable						
Fasting blood sugar						
Constant	83.4	9.4	8.9	0.000	65.02, 101.84	0.005
Birthweight	−5.1	1.8	−2.8	0.005	−8.61, −1.54	
Age	0.5	0.2	2.5	0.012	0.11, 0.89	
Gender	1.8	2.1	0.9	0.377	−2.26, 5.95	
Model 1: Dependent variable						
Blood pressure—systolic						
Constant	109.8	3.0	36.8	0.000	103.98, 115.70	
Birthweight	1.6	1.0	1.7	0.098	−0.29, 3.46	0.0983
Model 2: Dependent variable						
Blood pressure—systolic						
Constant	116.8	5.7	20.3	0.000	105.49, 128.10	0.551
Birthweight	0.7	1.1	0.6	0.551	−1.50, 2.80	
Age	0.1	0.1	0.5	0.649	−0.18, 0.29	
Gender	−4.6	1.3	−3.6	0.000	−7.14, −2.10	
Model 3: Dependent variable						
Blood pressure—systolic						
Constant	108.1	5.3	20.5	0.000	95.90, 117.70	0.485
Birthweight	−1.0	0.9	−1.0	0.485	−2.78, 1.32	
Age	−0.1	0.1	−1.2	0.278	−0.36, 0.10	
Gender	−5.9	1.2	−5.0	0.000	−8.19, −3.43	
BMI	0.9	0.1	7.7	0.000	0.64, 1.09	
Model 1: Dependent variable						
Blood pressure—diastolic						
Constant	73.4	2.5	29.5	0.000	68.50, 78.27	0.0679
Birthweight	1.5	0.8	1.8	0.068	−0.11, 3.02	
Model 2: Dependent variable						
Blood pressure—diastolic						
Constant	78.5	4.7	16.5	0.000	69.12, 87.78	0.441
Birthweight	0.7	0.9	0.8	0.441	−1.08, 2.47	
Age	0.1	0.1	1.1	0.292	−0.09, 0.30	
Gender	−4.4	1.1	−4.1	0.000	−6.45, −2.29	
Model 3: Dependent variable						
Blood pressure—diastolic						
Constant	71.2	4.4	16.0	0.000	62.03, 80.30	0.722
Birthweight	−0.3	0.8	−0.4	0.722	−2.03, 1.41	
Age	0.0	0.1	−0.3	0.784	−0.22, 0.17	
Gender	−5.3	1.0	−5.3	0.000	−7.24, −3.25	
BMI	0.6	0.1	6.7	0.000	0.45, 0.82	

BMI=Body mass index; CI=Confidence interval; Coeff.=Co-efficient; SE=Standard error

A significant positive association was observed between birthweight and BMI (p=0.007); additionally, adjusting for age and gender, the association was highly significant with BMI (p=0.000). Conversely, a significant negative association was observed between birthweight and adult levels of fasting plasma glucose (p=0.016), and after adjusting for age and gender, this was even more significant (p=0.005). No association was observed between birthweight and adult blood pressure: systolic (p=0.098) and diastolic (p=0.068). Even after adjustment for age, gender, and BMI, no significant association was observed between systolic and diastolic blood pressure and birthweight. The association between short and obese and adult variables was not different from those who were tall and obese

Data were also analyzed according to infant-feeding status. Grouping infants in predominantly-breastfed, partially-breastfed, or bottle-fed at three months of age showed no significant mean differences with adult variables (data not shown).

## DISCUSSION

More than 90% of the world's low-birthweight babies are born in developing countries, and the aetiology of many of these can still be attributed to poor maternal nutritional status. At the same time, many of these countries are undergoing a rapid nutritional transition with increasing affluence and the associated emergence of chronic disease. The implications of the ‘developmental origins of health and disease’ hypothesis are, therefore, of greatest public-health relevance in such countries, and opportunities to investigate these relationships are of importance in understanding the mechanisms of this hypothesis in more detail (9). Pakistan is one such transitional society where there is a high prevalence of foetal undernutrition (23,24) and an increasing prevalence of overweight and obesity in adulthood (11). The detailed data we have dating back to the 1960s and 1970s provided a rare opportunity to study the association between early life and later risk of disease in a cohort of urban adults born, and still living, in Lahore, Pakistan.

In this population group, the prevalence of foetal undernutrition was high, with 16% of the study subjects born of a low birthweight. When this population group was revisited during adulthood, a contrasting picture was observed, and using the proposed classification of BMI for Asians (22), 25% of the study subjects were overweight, and nearly 40% were either obese or very obese. This high level of overweight and obesity could be predicted to result in increased risk from a range of metabolic consequences (2,10).

In the present study, impaired glucose tolerance was observed in 6.2%, diabetes in 3.2%, raised systolic blood pressure in 6.4%, and raised diastolic blood pressure in 16.6% of the subjects. Higher levels are quoted for developing countries (25). The National Health Survey of Pakistan showed that, for men and women in Pakistan, hypertension increases with age ranging from a low of 3% for urban females aged 15–25 years to a high of 58% for urban female age-group of 65 years and above (10). In the male Pakistani population, the prevalence is 10% in the age-group of 15–25 years, and 35% in the age-group of 65 years and above (10). A diabetes prevalence of 11.8% is reported for the adult Pakistani population in the UNICEF report 2003 (26). These values are considerably higher than 2.7% observed in the current study subjects. This is likely to be a consequence of the comparatively younger age range of these subjects (mean=29 years). The observed prevalence of impaired glucose tolerance of 6.5%, coupled with the extremely high prevalence of overweight and obesity, could predict an increase in the prevalence of diabetes with age.

A negative association was found with fasting plasma glucose levels, a strong positive association was found with adult BMI, and no association was found with adult blood pressure. The suggestion of an inverse relationship between birthweight and plasma glucose levels is consistent with data from India, indicating that poor growth *in utero,* or during early infancy is a strong predictor of later glucose intolerance and diabetes risk (13). With increasing age in this urban Pakistani population, one might predict an amplification of this relationship. Possible reasons for the lack of an association between birthweight and blood pressure include the strong correlation between blood pressure and adult BMI negating any influences of the early-life environment. As observed by Schieri *et al.*, our study did not show any association between the short obese and adult variables compared to tall obese (27).

Using the data from the present study, we were unable to find any significant associations between rates of growth during infancy and later risk of chronic disease. It is possible that this is a consequence of the high rates of infant malnutrition and, hence, a comparatively low number of infants demonstrating accelerated growth between birth and later infancy and childhood. Singhal and colleagues have recently demonstrated an association between feeding of breast milk during infancy and an improved metabolic profile in adolescence (28), providing experimental evidence for the long-term benefits of breastfeeding on the risk of atherosclerosis. In a separate paper, the same authors hypothesized that rapid growth acceleration as a consequence of bottle-feeding could explain the observed associations among small size at birth, rapid catch-up growth, and risk of cardiovascular diseases in later life (29). We were also able to look at early infant-feeding status and risk of adult disease, but no association was observed between infant-feeding status and the selected risk factors for adult diseases, possibly because exclusive breastfeeding was non-existent in this cohort.

In relation to the observations of the present study, it is of interest to note that a subsample of subjects from this cohort have been used for exploring the hypothesis that immune function is programmed by events in early life. Using antibody response to vaccination, a significant positive association was observed between small size at birth and a reduced response to a polysaccharide typhoid vaccine (30). This finding could suggest that, in populations with a high prevalence of early malnutrition, followed by chronic exposure to a number of infectious pathogens, the development of the immune system is impaired by events early in life.

Limitations of the current study include: the small range of markers of chronic disease measured; the fact that birthweight was not always measured immediately after delivery; selection bias, in that this subsample from the original cohort does not reflect the whole cohort; or that the adults in this cohort are too young to detect any such association and that one might predict the emergence of disease with an increase in age, especially in view of their developing levels of overweight and obesity. Dyslipidaemia was not explored due to the high cost of the test. Despite these limitations, we have shown that small size at birth in Pakistani adults born in an urban slum is correlated with higher levels of fasting plasma glucose, suggesting that small size at birth, followed by adequate nutrition in adult life, may be a risk factor for later chronic disease. This study further highlights the importance of improving the nutritional status of women of reproductive age, especially in populations undergoing rapid economic transition.

## ACKNOWLEDGEMENTS

The Government of Pakistan financed the original cohort study, and the Nestlé Foundation financed the follow-up study. The authors are grateful to both.

## References

[1] Barker DJ (1995). Fetal origin of coronary heart disease. BMJ.

[2] Barker DJP, Gluckman PD, Godfrey KM, Robinson JS (1993). Fetal nutrition and cardiovascular disease in adult life. Lancet.

[3] Law CM, Shiell AW (1996). Is blood pressure inversely related to birth weight? The strength of evidence from a systematic review of the literature. J Hyperten.

[4] (1998). Report of Expert Committee on Diagnosis and Classification of Diabetes Mellitus. Diabet Care.

[5] Charles NA, Fontboune A, Thibult N, Warnet JM, Rosselin GE, Esehwege E (1991). Risk factors for NIDDM in white population. Paris propective study. Diabetes.

[6] Desai M, Crowther NJ, Ozann SE, Lucas A, Hales CN (1995). Adult glucose and lipid metabolism may be programmed during fetal life. Biochem Soc Trans.

[7] Prentice MA (2001). Obesity and its potential mechanistic basis. Br Med Bull.

[8] Moore SE, Halsall I, Howarth D, Poskitt EM, Prentice AM (2001). Glucose, insulin and lipid metabolism in rural Gambians exposed to early malnutrition. Diabet Med.

[9] Adair LS, Prentice AM (2004). A critical evaluation of fetal origins hypothesis and its implication for developing countries. J Nutr.

[10] (1994). Pakistan Medical Research Council. National health survey of Pakistan.

[11] Misra A, Pandey RM, Devi JR, Sharma R, Vikram NK, Shanna N (2001). High prvalence of diabetes, obesity and dyslipidaemia in urban slum population in northern India. Int J Obes Relat Metab Disord.

[12] Yajnik CS, Fall CH, Coyaji KJ, Hirve SS, Rao S, Barker DJ (2003). Neonatal anthropology: the thin-fat Indian baby. The Pune Maternal Nutrition Study. Int J Obes Relat Metab Disord.

[13] Yajnik CS (2004). Early Life origins of insulin resistance and type 2 diabetes in India and other Asian countries. J Nutr.

[14] Jalil F (1974). Factors adversely and favourably affecting health of children birth—2 years.

[15] (1995). World Health Organization. Physical status; use and interpretation of anthropometric indicators of nutritional status.

[16] Jalil F, Lindblad BS, Hanson LA, Khan SR, Yaqoob M, Karlberg J (1993). Early child health in Lahore, Pakistan: IX. Perinatal events. Acta Paediatr.

[17] Neita RT, Elving LD, Luttermann JA (2002). Accurate measurement of arterial blood pressure, body position and blood pressure measurement in patients with diabetes mellitus. J Intern Med.

[18] Tietz NW (1995). Clinical guide to laboratory tests.

[19] Chatterjee S, Hadi AS (1986). Influential observation, high leverage points, and outliers in linear regression. Stat Sci.

[20] Cook RD (1977). Detection of influential observations in linear regression. Technometrics.

[21] Karlberg J, Ashraf R, Saleemi M, Yaqoob M, Jalil F (1993). XI. Growth. Early child health in Lahore, Pakistan. Acta Peadiatr Scand.

[22] (2004). WHO Expert Consultation. Appropriate body mass index for Asian populations and its implication for policy and intervention strategies. Lancet.

[23] Arif MA, Nizami SQ (1985). Study of 10566 newborn babies. Pakistan Paediatr J.

[24] (2001). Newborn health. State of the world's newborns: Pakistan.

[25] Law CM, Egger P, Dada O, Delgado H, Kalberg E, Lavin P (2001). Body size at birth and blood pressure among children in developing countries. Int J Epidemiol.

[26] (2003). Eastren Mediterranean Region; regional features.

[27] Schieri R, Santos SK, Alves PR, Ascherio A (2000). Short stature and hypertension in the city of Rio de Janerio, Brazil. Public Health Nutr.

[28] Singhal A, Cole TJ, Fewtrell M, Lucas A (2004). Breast milk feeding and lipoprotein profile in adolescent born pre-term: follow-up of prospective randomized study. Lancet.

[29] Singhal A, Lucas A (2004). Faster growing children are at a greater risk of heart disease and stroke. Lancet.

[30] Moore SE, Jalil F, Ashraf RN, Szu SC, Prentice AM, Hanson LA (2004). Birth weight predicts response to vaccination in adults born in an urban slum in Lahore, Pakistan. Am J Clin Nutr.

